# Planning Marine Reserve Networks for Both Feature Representation and Demographic Persistence Using Connectivity Patterns

**DOI:** 10.1371/journal.pone.0154272

**Published:** 2016-05-11

**Authors:** Michael Bode, David H. Williamson, Rebecca Weeks, Geoff P. Jones, Glenn R. Almany, Hugo B. Harrison, Jess K. Hopf, Robert L. Pressey

**Affiliations:** 1 ARC Centre of Excellence for Environmental Decisions, School of Botany, The University of Melbourne, Parkville, Melbourne, VIC, 3010, Australia; 2 Australian Research Council Centre of Excellence for Coral Reef Studies, James Cook University, Townsville, 4811, QLD, Australia; 3 College of Marine and Environmental Sciences, James Cook University, Townsville, 4811, QLD, Australia; 4 Centre National de la Recherche Scientifique-EPHE-UPVD, Universite de Perpignan, 66860, Perpignan Cedex, France; Biodiversity Research Center, Academia Sinica, TAIWAN

## Abstract

Marine reserve networks must ensure the representation of important conservation features, and also guarantee the persistence of key populations. For many species, designing reserve networks is complicated by the absence or limited availability of spatial and life-history data. This is particularly true for data on larval dispersal, which has only recently become available. However, systematic conservation planning methods currently incorporate demographic processes through unsatisfactory surrogates. There are therefore two key challenges to designing marine reserve networks that achieve feature representation and demographic persistence constraints. First, constructing a method that efficiently incorporates persistence as well as complementary feature representation. Second, incorporating persistence using a mechanistic description of population viability, rather than a proxy such as size or distance. Here we construct a novel systematic conservation planning method that addresses both challenges, and parameterise it to design a hypothetical marine reserve network for fringing coral reefs in the Keppel Islands, Great Barrier Reef, Australia. For this application, we describe how demographic persistence goals can be constructed for an important reef fish species in the region, the bar-cheeked trout (*Plectropomus maculatus*). We compare reserve networks that are optimally designed for either feature representation or demographic persistence, with a reserve network that achieves both goals simultaneously. As well as being practically applicable, our analyses also provide general insights into marine reserve planning for both representation and demographic persistence. First, persistence constraints for dispersive organisms are likely to be much harder to achieve than representation targets, due to their greater complexity. Second, persistence and representation constraints pull the reserve network design process in divergent directions, making it difficult to efficiently achieve both constraints. Although our method can be readily applied to the data-rich Keppel Islands case study, we finally consider the factors that limit the method’s utility in information-poor contexts common in marine conservation.

## Introduction

The exchange of individuals among patches of spatially-discrete habitat (“connectivity”) has broad implications for how and whether species persist in a region, how they respond to natural and anthropogenic disturbances at both ecological and evolutionary timescales [1,2], and how they should be managed [3–5]. Connectivity contributes to the persistence and dynamics of metapopulations [6,7], and the structure of metacommunities [8–10], through replenishment of local populations and post-disturbance recovery. Connectivity is especially important in the marine environment, where almost all fish and invertebrate species have an obligate and extended pelagic larval phase [11,12], and strong ocean currents can carry dispersing larvae long distances [13–15]. Because of its central role in the life-cycle of reef fishes, connectivity should be considered when designing networks of marine reserves [16–20]. Marine reserves, particularly no-take areas, only constitute a relatively small proportion of important habitats [21], even in the best protected habitats such as the Great Barrier Reef Marine Park in Australia [21]. Because reef fish populations outside marine reserves are generally depleted [22], and in some contexts (e.g., the Philippines) almost non-existent [23], connectivity is required for these separated protected areas to exchange enough larvae to support persistent populations, and also to provide the spillover that exports their benefits to the broader, unprotected landscape [24].

Marine reserve networks must therefore satisfy two constraints simultaneously. First, they should guarantee the representation of key conservation features in the reserve network. This is a primary goal of all systematic conservation planning [25]. Important facets of biodiversity such as species, habitat types, and ecological processes should occur within no-take reserves, somewhere in the network (a “feature representation constraint”). Second, the networks should ensure that sufficient larvae are being exchanged between populations to ensure that those populations will persist into the future (a “demographic persistence constraint”) [18,26]. This requires connectivity to be incorporated explicitly. While understanding the process of larval connectivity is critical to the success of marine reserve networks [4,27], most systematic conservation planning theory has focused only on feature representation, with a range of planning tools available to implement these theories [25,28]. However, these tools do not currently include persistence constraints; as a result, while the tools represent conservation features, it is unknown whether those features will be able to persist. In fact, there is reason to believe that reserve networks which only target representation constraints will fail to achieve persistence constraints. Potential reserve sites that are further apart are likely to exhibit greater differences in species composition, and therefore to be selected in an efficient complementary reserve system. However, such sites are less likely to be demographically connected (and are therefore less likely to persist) because dispersal strength diminishes rapidly with distance [26,29,30].

Planning for marine connectivity has historically been constrained by a lack of modelled or empirical data. In-situ observations of larval dispersal are almost impossible, because the density of dispersing larvae in the planktonic environment is vanishingly small [5,31]. Nevertheless, in the last decade, quantitative larval dispersal data have become available for an increasing number of species and locations, at ever greater spatial scales and higher resolutions. Some of this information has come from population genetics [32,33] and biophysical modelling [13,34,35], which have estimated larval connectivity over very broad spatial and temporal extents [17,36–39]. Simultaneously, the recent emergence of methods for genetic parentage [24,40,41] and otolith microchemistry [40,42] have begun to provide empirical dispersal data across spatial and temporal scales that are relevant to spatial management planning. Unfortunately, this new dispersal data has revealed fundamental limitations to conservation planning theory.

Marine resource managers want to understand connectivity because they recognize that, unless connectivity can be maintained, the ongoing persistence of key conservation features cannot be guaranteed. However, most conservation planning methods cannot incorporate dynamical processes such as connectivity. Systematic conservation planning theory calculates the performance of potential protected area locations via data on features and processes [25,43]. To date, these methods have almost exclusively used static data on biodiversity, such as the location of habitat types, or static species distribution models [44–46]. Acknowledging the importance of dynamical processes, researchers have made a series of modifications to their standard methods for planning that allow them to include connectivity. These modifications fall into four categories; for a variety of reasons, each is unsatisfactory. The most common category applies quantitative “rules of thumb” for MPA size and spacing [47,48]. While straightforward and often derived from empirical data on connectivity, these rules involve overly simplistic assumptions that ignore spatial heterogeneity in habitat availability and dispersal patterns. The second approach uses connectivity patterns to rank habitat patches, generally using metrics from network theory (e.g., centrality, or eigenvalue analysis; [49–53]). While these metrics make some intuitive sense, they have no clear ecological or demographic interpretation [54]. It is therefore unclear if the resulting reserve networks ensure persistence. The third approach ranks planning units using connectivity directly, but focuses on only a small subset of the data, such as the self-recruiting proportion [52,53], even though metapopulation persistence depends on dispersal between populations [13,55]. The fourth approach is to treat connectivity as a feature that requires representation in the reserve system, similar to species occurrences [56–58], including widely used methods such as Marxan’s boundary-length modifier [59]. Doing so makes the unreasonable assumptions that connectivity can be traded-off within and between species, and that lower connectivity can be tolerated if the reserve network is cheaper or larger. Such approaches also treat connectivity as a fundamental conservation objective, rather than a means of ensuring that species persist. Because none of these four approaches incorporate connectivity into an explicit demographic process, the performance of the resulting reserve networks (and thereby the underlying approaches) must be assessed *post hoc* using population viability analyses that explicitly evaluate persistence [50,52,53]. Such assessments are not common however, and even if their results provide indirect validation of an approach, a more direct inclusion of connectivity into the life-cycle of the species of interest is still preferable. *Post hoc* validation also cannot explain what aspect(s) of indirect approaches to connectivity planning were successful or not in achieving persistence.

A wide range of tools and methods are available to ensuring reserve networks are representative [28]. However, there are two main challenges to correctly incorporating connectivity alongside representation. First, from an ecological perspective, managers need to be able to translate the new data on larval dispersal into quantitative expressions of how much dispersal is needed to guarantee demographic persistence in each planning unit. For coral reef fishes, these requirements will need to be based on the full complexity of the larval connectivity patterns (i.e., not just self-recruitment), but also critical post-recruitment demographic processes such as mortality and reproduction—connectivity patterns aren’t enough by themselves. Second, from the perspective of conservation planning, methods are needed that include constraints for demographic persistence, and then integrate them with the representation constraints of multi-feature conservation planning. To address these challenges, we describe a new analytical method for designing MPA networks that satisfy the dual constraints of feature representation and—via connectivity patterns—the persistence of species with obligate larval dispersal phases.

## Methods

We begin by defining the planning method for a general conservation seascape (i.e., a marine landscape) that contains an arbitrary number of conservation features that need to be represented, and also a set of target species whose persistence needs to be guaranteed. These features and species are distributed among a set of planning units. We then demonstrate the application of this method by parameterizing it for a study area—the Keppel Islands group within the Great Barrier Reef Marine Park, Australia. This case study shows how the general model is parameterized, in particular how recruitment constraints can be defined.

### Management constraints and the objective function

In a seascape (i.e., a marine landscape) of *P* planning units (reefs, sections of coastline, etc), marine reserve networks are defined by the binary vector **N**, whose elements *N*_*i*_ indicate whether planning unit *i* is protected (*N*_*i*_ = 1) or unprotected (*N*_*i*_ = 0). Managers need to choose a reserve network that will equal or exceed two separate sets of constraints, while maximising an objective function. The first constraint ensures that a sufficient amount of each important conservation feature is found within the reserve network (the feature representation constraint). The second constraint ensures that every target species, in each protected or unprotected patch, receives enough recruitment to replenish populations and maintain persistence at a metapopulation scale (the demographic persistence constraint). Our method satisfies these two constraints while minimising a network performance metric: the reduction in fishing opportunities resulting from the no-take reserves.

#### Constraint 1: Represent conservation features within the marine reserve network

Each planning unit contains a known amount of each feature of conservation interest (total of *F* features, e.g., habitat, ecological processes or species), stored in a (*P* × *F*) matrix denoted **M**. Managers define constraints *Q*_*k*_ that correspond to each conservation feature *k*. An adequate reserve network will protect a set of planning units such that the total amount of each feature protected equals or exceeds its respective representation constraint.

$$\overset{P}{\underset{i=1}{\sum }}N_{i}\cdot {\left[M\right]}_{i,k}\geq Q_{k},\;\;\;\forall k.$$ (1)

#### Constraint 2: Ensure the demographic persistence of key species

Each planning unit hosts populations of *S* non-interacting species that experience fishing mortality, either as target species or bycatch, and whose dynamics can be described using metapopulation models. The species all have sessile life-history strategies, that is, adults do not move between distinct reef patches, but populations are demographically connected via the dispersal of larvae. For each species *s*, the amount of dispersal between each planning unit is defined by two *P* × *P* recruitment matrices, $R_{s}^{*}$ and **R**_*s*_. The element on row *i* and column *j* in each recruitment matrix represents a number of dispersing juveniles produced in planning unit *i* that would survive dispersal to settle and recruit in planning unit *j*. The first matrix, ${\left[R_{s}^{*}\right]}_{ij}$, states the recruitment that will occur when the source patch, *i*, is unprotected, the second, [**R**_*s*_]_*ij*_, states the additional recruitment (i.e., above the level offered by an unprotected source) that would occur if that source population were protected. For the case study that follows, we define the recruitment matrices using dispersal kernels, but note that they can be defined by any description of connectivity, and that they can be asymmetric, contain large-scale structure, or not be strongly connected [15,60–63].

To satisfy the demographic persistence constraint—to ensure that the population does not decline to zero—managers must ensure sufficient ongoing larval recruitment. We assume that this outcome can be achieved if the recruitment to each planning unit *i*, both protected and unprotected, equals or exceeds the natural rate of mortality. If the marine reserve network can achieve this constraint, then populations in protected planning units will persist, since well-enforced no-take reserves will only experience natural mortality. On unprotected planning units, this level of recruitment will not be sufficient to maintain fished populations at the same densities as in reserves. However, it will be sufficient to maintain persistent sink populations in unprotected planning units, and therefore to support ongoing catches. Calculating this constraint *T*_*i*,*s*_ is therefore equivalent to calculating natural mortality rates and the recruitment needed to replace it. We illustrate how this is done for the Keppels case study below.

The demographic persistence constraint is expressed as:

$$\sum_{i=1}^{P}\left(N_{i}{\left[R_{s}\right]}_{ij}+{\left[R_{s}^{*}\right]}_{ij}\right)\geq T_{j,s},\;\;\forall j\forall s.$$ (2)

The second term in the summation (the recruitment from unprotected planning units) does not depend on the control values *N*_*i*_ and is a constant. It could therefore be moved to the right hand side of the inequality. This would change the constraint from a minimum total recruitment, into a minimum additional recruitment coming from inside the reserve network. We also note that we can incorporate the common “scorched-earth” assumption (where populations on unprotected planning units are equal to zero) into our model by adding $R_{s}^{*}$ to **R**_*s*_, and replacing $R_{s}^{*}$ with zeros. Finally, while this formulation assumes that connectivity is constant through time, the basic form of Eq (2) can be modified to incorporate temporal variation in larval dispersal patterns [13]. If connectivity matrices from different years are incorporated into the constraint as pseudo-species, the resultant reserve network would satisfy the demographic persistence constraints for every year in the dataset.

The demographic persistence constraints will generally vary with the identity of the planning unit *i*. These factors can therefore reflect spatial heterogeneity in the habitat quality of planning units, such as different rates of mortality, different population densities, or different reproductive rates. In terrestrial population viability analyses, such factors are often derived from correlative ecological niche models [64], or spatial ecophysiological models [65], although this is not as common for marine species. The elements of the recruitment matrices are also able to vary, depending on both the output of the source reefs, and the potentially heterogeneous larval dispersal patterns between the planning units. Unprotected reefs will produce fewer recruits in our analyses since they support fewer reproducing adults, but output can also vary with the quality of the source habitat, or the direction and strength of ocean currents [15]. The formulation of Eq (2) ignores transient dynamics and therefore the constraints will only be guaranteed once the reserve network has been in place for time periods that are longer than the generation times of the focal species, and assuming that the system has not passed any irreversible abundance constraints.

#### Objective function: Minimize forgone fishing

We assume that the protection of any planning unit (*N*_*i*_ = 1) will have a negative impact on local extractive activities that can be decomposed into two multiplicative factors. These are the abundance of each species found in each planning unit when it is fished, *b*_*i*,*s*_, and the value of an individual of species *s* to the fishers, relative to the most valuable species, *V*_*s*_. Management wants to satisfy the conservation constraints while minimizing the sum of this impact across the network. Therefore, the managers’ objective is to minimize:

$$\underset{N}{\text{min}}\overset{P}{\underset{i=1}{\sum }}N_{i}W_{i},$$ (3)

where $W_{i}=\Sigma_{s=1}^{S}V_{s}b_{i,s}$: essentially the value of each planning unit. Eq (3) does not take into account the potential responses of fishers to the closure of fishing grounds [66,67]. The likely impact of implementing reserves is an increase in the intensity of fishing effort on unprotected planning units, the so-called “squeeze effect” of displaced effort. If the fishery was not overfished before reserves were established, this displaced effort on unprotected reefs can result in lower spawning stock biomass, reduced harvests, and higher per-unit extraction costs. This would make the absolute value of our objective function an underestimate of fishery impacts, although it may not alter the relative performance of different reserve networks.

### Finding the optimal solution

Each of the elements in this problem—the performance metric and both constraints—are linear, and the optimal solution to this problem can therefore be found by applying binary integer programming methods [28]. We describe in detail how the above problem can be thus formulated in S1 Text.

### Case study: Keppel Island group, Great Barrier Reef Marine Park

**Fig 1:**
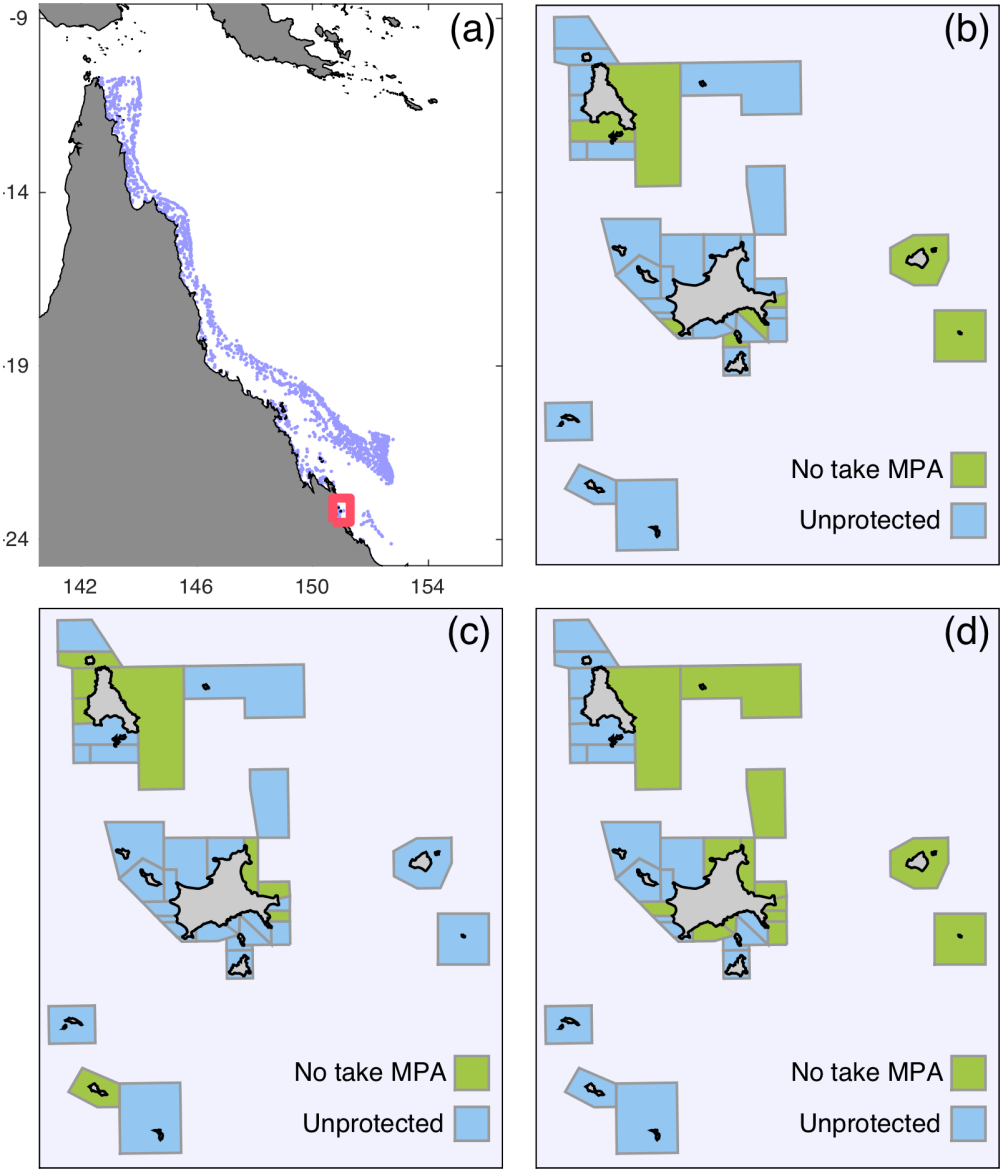
(a) Location of the Keppel Islands on the east coast of Australia. Individual reefs in the Great Barrier Reef are shown with light blue markers. The red box indicates the specific location of the Keppel Islands in the southern GBRMP. The remaining figures show the detailed location of the Keppel Islands and different optimal reserve networks. Colored polygons indicate the planning units: no-take reserve in green, non-reserve in blue. (b) The reserve network that satisfies constraints for both feature representation and demographic persistence, while minimizing the opportunity costs to fishers. (c) The reserve network that satisfies the feature representation constraints at a minimum cost to fishers. (d) The reserve network that satisfies the demographic persistence constraints at a minimum cost to fishers. The map incorporates data which is the copyright of the Commonwealth of Australia (Great Barrier Reef Marine Park Authority), and used with permission of the Commonwealth. The Commonwealth has not evaluated to Data as altered and incorporated, and therefore gives no warranty regarding its accuracy, completeness, currency or suitability for any particular purpose.

The Keppel Islands group is an archipelago of high continental islands off the central Queensland coast, within the Great Barrier Reef Marine Park (GBRMP) (Fig 1). The fringing coral reefs support large populations of bar-cheek coral trout (*Plectropomus maculatus*), which is one of the key target species of the reef line fishery operating in the GBRMP [68]. The species is also a favourite species of local recreational fishers, and has been the focus of recent intensive research on its life history and dispersal abilities [24,69,70]. Approximately 700 ha of fringing coral reefs within the island group are protected by a multiple-use zoning management plan that includes areas that are open to fishing (recreational and commercial), and a network of limited-use and no-take reserve areas [21]. Since the rezoning of the GBRMP in July 2004, approximately 28% (196 ha) of the reef area within the Keppel Island group has been protected within no-take reserves. To demonstrate a hypothetical application of our general method, we devised a *de novo* reserve network for this island group, concentrating on protecting three habitat features and ensuring the persistence of *P*. *maculatus*.

#### Feature representation constraints

We began by defining 36 planning units across the region (Fig 1b–1d). Some planning units contain a single patch reef, some contain a part of a larger reef or island, and some contain multiple small patch reefs. These 36 planning units align with the current zoning plan for the Keppels region, which includes three coral reef management zones: Habitat Protection Zones (HPZ); Conservation Park Zones (CPZ); and Marine National Park (no-take) Zones (MNPZ) [71]. Due to the close proximity to the mainland and ease of access, fringing reefs in the Keppel Islands are almost exclusively fished by recreational fishers, for whom HPZ and CPZ reefs are open to fishing (Williamson et al. 2014). Our planning method consequently considers these two zones to be unprotected (*N*_*i*_ = 0), and MPNZs to be protected (*N*_*i*_ = 1).

The reefs in the Keppels are entirely classified as bioregional type RE8: Coastal Southern Fringing Reefs, a bioregion that also includes all near shore fringing reefs to the north and south of the Keppels group, but not the Capricorn Bunker reefs, 70 km to the southeast. For this higher resolution exercise, we further split reef habitat into three strata—reef flat, reef crest, and reef slope. These three habitat features differ significantly in their exposure to light and exposure [72], and consequently have substantially different species assemblages, particularly of coral species [73,74]. We therefore aim to represent each of these habitat types within our MPA network. Constraints for representation are set at 35% of each habitat type within the Keppel Island group, reflecting the goals of the 2004 GBR rezoning. However, we acknowledge that the target amount of habitat representation is a difficult socio-ecological question that may differ between the Keppel group and the GBR as a whole. Data on the distribution of habitat types were calculated using ground-truthed satellite imagery in ArcGIS. The resulting feature matrix **M** is in S1 Table.

#### Demographic persistence constraints

The following section details how we choose constraints for annual recruitment into each planning unit (*T*_*i*,*s*_) which ensure that all protected planning units receive sufficient recruitment to exceed or equal natural mortality rates. Unprotected reefs receive at least the same level of recruitment, which will sustain ongoing catches (though not pristine densities). Self-recruitment will be a key component of this recruitment, and is included in our models. However, the estimated scale of larval dispersal [29], and observed dispersal between marine reserves [24], strongly suggest that adequate recruitment will require immigration from other reefs. Our method takes both sources of recruitment into account, using the recruitment matrices to measure their relative contribution.

Based on a von Bertalanffy growth curve parameterized for *P*. *leopardus* (a closely-related sister-species; [75]), we estimate that *P*. *maculatus* individuals in the first-year class (from recruitment to age one) have a fork length of ≤ 25 cm. Surveys of the abundance and size of the species on the slope habitat of existing protected reefs report an average of 46 such individuals per hectare. These 46 individuals are the survivors of larger settling cohorts, and to convert this density into a recruitment target for each planning unit, we correct for two different forms of mortality. First, recruits experience a very high rate of mortality in the first 48 hours post-settlement, estimated at 56% [76]. Following this, a study estimated the average ongoing mortality rate of *Plectropomus* spp. recruits in their first year at 60% [77], which is equivalent to a daily mortality rate of 0.25%. Assuming that larvae arrive at a constant rate throughout the year, these mortality rates imply that the 46 surviving individuals per hectare in the first year age class are the result of 160 settlers/hectare of slope habitat annually.

We adapt this constraint to create values for *T*_*i*,*s*_ in each planning unit. The density of reproductively mature female adults in each habitat type, in protected reefs, was estimated using habitat stratified counts carried out throughout the Keppel Islands in 2008, which estimated the density and length-frequency distributions of all adult *P*. *maculatus* individuals. On the reef crest, slope and flat, adult densities are 48, 99 and 26 individuals per hectare, respectively. Since each habitat type exhibits similar size structures, we use these relative densities to calculate recruitment constraints of 78, 160 and 42 settlers per hectare of crest, slope and flat. As an example of how the different *T*_*i*,*s*_ values are calculated from these densities, the planning unit that surrounds Barren Island contains 13, 26.2, and 1.9 hectares of reef crest, slope and flat respectively (see S1 Table). We therefore want to create a reserve network that will deliver a total of 5,277 larvae to the Barren Island planning unit.

#### Recruitment data (R_*s*_)

Empirical data and biophysical modelling have demonstrated significant levels of larval retention and exchange for *P*. *maculatus* within and among reefs in the Keppel Island group [24]. The destination of larvae that are spawned within each planning unit is determined by a combination of oceanographic influences and larval behavior. Oceanography is driven by a strong local tidal regime, the broader Mackay macro-tidal regime, seasonal wind-driven connectivity with the larger Capricornia group of reefs, and episodic events of the southward-flowing East Australia Current. *P*. *maculatus* larvae have relatively long pelagic larval durations of 24–29 days [24]. Larvae are thought to be released in small-group spawning aggregations that occur between October and March inclusive [78], for five days either side of new moons.

We estimated this dispersal using recruitment matrices **R**_*s*_ and $R_{s}^{*}$, based on data gathered for *P*. *maculatus* in the Keppels region, where a large parentage assignment experiment sampled juveniles and adults from across the Keppels between 2007–2009 (Harrison *et al*. 2012). Parentage samples could not be used directly to create recruitment matrices because only a subset of reefs were sampled, and because parentage could only be assigned to a subset of sampled recruits. To extrapolate this data across all planning units, we therefore fit larval dispersal kernels to the data [79]:

$$p_{ij}=\frac{1}{2\pi \phi^{2}}\text{exp}\left[-\frac{d_{ij}}{\phi }\right].$$ (4)

Where the Euclidean distance between reefs is denoted *d*_*ij*_, and is used to predict the probability *p*_*ij*_ that a larvae spawned at planning unit *i* would disperse and settle on planning unit *j*, conditional on it surviving the larval phase. The parameter *ϕ* is estimated at 12.38 for the Keppels. We transform these proportions into the number of settlers by multiplying *p*_*ij*_ by the total number of larvae produced annually in each planning unit *i*. The number of larvae that travel from unprotected planning unit *i* and recruit to any planning unit *j* (i.e., protected or unprotected) is therefore:

$${\left[R_{s}^{*}\right]}_{ij}=\theta \;p_{ij}\overset{3}{\underset{h=1}{\sum }}\overset{15}{\underset{y=1}{\sum }}f_{y}\;a_{i,h}\;\phi_{y}\;\rho_{y,s,h}^{*}$$ (5)

where *f*_*y*_ is the fecundity of a female of length class *y*. Each length class is 5 centimetres, with the maximum length observed equal to 75 centimetres; the length of each class is estimated using the upper bound of the class, designated *l*_*y*_. *ϕ*_*y*_ is the proportion of adults in length class *y* who are female, and *θ* is the proportion of spawned larvae who die during the dispersal phase. The variables $\rho_{y,s,h}^{*}$ represent the density of adults of species *s* and length *y* in habitat *h* on unprotected reefs (*ρ*_*y*,*s*,*h*_ gives the density on protected reefs), and *α*_*i*,*h*_ is the amount of habitat of type *h* in planning unit *i* (*h* is either 1: crest, 2: slope, or 3: flat). We can calculate [**R**_*s*_]_*ij*_ by substituting ($\rho_{y,s,h}-\rho_{y,s,h}^{*}$) for $\rho_{y,s,h}^{*}$ in Eq (5). For the remainder of this section, we go through our process of estimating each of these parameters. Note that, while our probabilities of dispersal are symmetrical (i.e., *p*_*ij*_ = *p*_*ji*_), the recruitment matrices are not, since the source populations are different (S1 Fig).

In the Keppel Islands, all reef habitats are defined as reef crest, slope or flat (the area *α*_*i*,*h*_, of each is the same as in the feature matrix). Each of these habitat types supports a different adult density, but approximately the same age-distribution (Kolmogorov-Smirnov test, *α* = 0.01). The density of reproductively mature adults of a given length *ρ*_*y*,*s*_ on protected reefs is estimated using habitat stratified counts as described in the recruitment section above. Their relative densities in protected and unprotected planning units were estimated in 2009, when a set of surveys was undertaken at 22 monitoring sites, on both reserve (MNPZ) and non-reserve (HPZ, CPZ) reefs. The results indicate that mean adult coral trout density is 1.8 times higher on reserve reefs than on non-reserve reefs. We assume that this proportional difference in densities between reserve and non-reserve reefs is consistent between the three habitat types, which themselves have different densities (already described). As a consequence of the different habitat distributions in each planning unit, the elements of the recruitment matrix vary greatly, reflecting both the habitat quality in the different planning units, their protected status, and the distance to the nearest larval destinations. Large reefs with higher proportions of slope habitat, that are both protected and close to other planning units, have the potential to operate as demographic sources in the metapopulation, and will therefore be prioritised for protection by the optimisation algorithm.

A changing proportion *ϕ*_*y*_ of adults are female, since the species is a protogynous hermaphrodite. The sex ratio of the individuals in different length classes was inferred from previous studies of the size-sex structure of *P*. *leopardus*, another protogynous hermaphrodite [80]. This study showed that *P*. *leopardus* in the central GBR begin to transition from female to male at a length of 32cm, and are exclusively male at lengths above 52cm. We therefore model the female proportion of the population as a linear relationship with length:

$$\phi_{y}=\{\begin{array}{cc} 1 & \text{if }y<7\\ \frac{x-7}{4} & \text{if }7\leq y\leq 11\\ 0 & \text{if }y>11 \end{array}$$ (6)

For *P*. *maculatus* we estimate the fecundity *f*_*y*_ using published allometric relationships for species in the genus *Plectropomus* that link length and fecundity [81]:

$$f_{y}=13.82{\left(l_{y}\right)}^{3.03}.$$ (7)

The larval mortality proportion *θ* is a very difficult component to parameterize. Larval mortality is thought to occur chiefly through predation, and literature estimates of the rate vary between 2% and 97% per day [82]. Given this uncertainty, we choose a value for *θ* that can recreate the adult abundance currently observed in the Keppels, consistent with our model of the metapopulation. Using the surveyed estimates of adult density (*ρ* and ρ*), the location of the current marine reserves in the Keppels, and estimates of larval dispersal derived from the best-fit kernels (*p*_*ij*_), we vary the value of *θ* until all of the current MNPZ zones received sufficient recruitment to justify their current populations (as described above, 78, 160 and 42 settlers per hectare of crest, slope and flat). These values imply that a mortality parameter of *θ = 7* × 10^−4^ will recreate the observed recruitment densities, a daily mortality rate of 26%. See S1, S2 and S3 Tables for recruitment data used.

#### Objective function

The recreational fishing community is the largest and arguably the most politically powerful stakeholder in the GBRMP, and were an influential voice during the 2004 rezoning of the GBRMP [83,84]. They are particularly important in the Keppel Islands, where the vast majority of the fishing effort applied to the reefs is from the recreation sector. The consumptive interest of recreational fishers in the GBRMP is focused on the number and size of fish caught during trips, with a premium placed on catching the mandated daily limits (bag limits) of large fish, and an additional aversion to trips that catch nothing [85]. Once the constraints for representation and persistence are satisfied, we assume that the primary concern of managers is to minimize the aggregate opportunity cost of the no-take reserves on recreational fishers. Specifically, to minimize the total number of fish that are no longer accessible to fishers because of the location of the no-take reserves.

We only consider one fish species, so we set *V*_1_ = 1 without loss of generality. We base the value of each planning unit on the number of adult individuals longer than the length restriction that exist on that planning unit when it was fished (≥ 38 cm total length) [86]. The total number of legal sized *P*. *maculatus* in each planning unit is estimated using the same 2009 habitat-stratified count data described above. The objective function therefore takes into account the relative suitability of the different habitat types. An alternative objective function based on lost access to coral trout biomass would also be straightforward to implement using these surveys and published allometric relationships between length and weight [17].

#### Analyses

For each of the analyses listed below, we record the time required to identify the optimal solution of the integer programming problem on a desktop computer, and report the run time and estimates for larger systems. Our analyses revolve around no-take reserve networks constructed using three different approaches. We begin by calculating a base scenario: a reserve network that meets dual constraints—both feature representation and demographic persistence—while minimizing impacts on recreational fishers. We then contrast this reserve network with a network that focuses solely on habitat representation, and then one aimed at demographic persistence only. We contrast these three networks based on their efficiency (i.e., the total proportion of reef area that must be protected to satisfy the constraints) and their degree of spatial overlap. The proportional overlap of two reserve networks is a commonly calculated measure of their spatial similarity [87], and we use it to assess the degree to which the three networks are protecting the same planning units. However, the amount of observed overlap must be compared to random expectation, since any two networks chosen at random will likely exhibit some overlap. Thus, we compare the proportional overlap of the reserve areas created using the three different approaches, to the amount of overlap observed within a set of 10,000 reserve networks, each made up of a comparable number of randomly selected planning units.

We further assess the degree to which persistence constraints were complementary to representation constraints. To do this, we calculate how the amount of area protected by a feature representation network needs to increase if managers want to satisfy the demographic constraints without explicitly planning for them. If planning units contributing highly to both representation and recruitment overlap, then recruitment constraints may require only small additions to the marine reserve network (or no additions at all). However, if planning units important for representation and recruitment are not congruent, the required increase in the coverage of no-take reserves could be substantial.

#### Sensitivity analyses

As we emphasise during our parameterisation of the Keppels case study above, many of the ecological parameters in these analyses are challenging to estimate. This is particularly true for the larval dispersal parameters (e.g., the daily rate of pelagic larval mortality), which are difficult to observe directly. Moreover, the feature representation and demographic persistence constraints are also difficult to define with confidence, particularly since they involve a combination of difficult empirical questions (e.g., what is the relative spawning stock biomass on reserved and fished reefs?) and complicated value-judgements (e.g., what level of coral reef degradation is the community willing to tolerate?). We consequently undertake a sensitivity analysis for our Keppels case study, focusing on the effects of uncertainty in both the ecological parameters, and the size of the constraints.

We vary four uncertain elements of the formulation—two different parameters, and the two constraints. In each test, we assess how robust the optimal reserve design is to pessimistic uncertainty. Instead of assessing whether the reserve design changes with a single arbitrary amount of uncertainty (e.g., within *x* = 5%), we calculate how much uncertainty the optimal network can tolerate before it fails to achieve both its demographic and feature representation constraints. The tests are: (1) we randomly increase each of the feature constraint levels (*Q*_*k*_) within ±*x*% of their original values. This corresponds to uncertainty about how much of a given feature needs protection to ensure it can persist into the future. (2) We alter each demographic recruitment constraints (*T*_*i*,*s*_) within ±*x*% of its original value, to reflect our uncertainty about how much recruitment is needed to maintain persistent populations on protected reefs. This is a particularly important sensitivity test to undertake, given the challenge of estimating these constraints. (3) We decrease the amount of each habitat type in each planning unit by within *x*% of its nominal value. The distribution of habitat across planning units is based on ground-truthed satellite data, but will still contain error at the high resolution of these analyses. (4) Finally, we decrease each element in the two recruitment matrices (**R**_*s*_ and $R_{s}^{*}$) by within *x*%. This final sensitivity test, which varies values in the recruitment matrices, can represent uncertainty in a wide range of ecological parameters, including larval mortality or pre-capita fecundity (see Eq 5). We only consider pessimistic uncertainty in the final two sensitivity analyses, since we are calculating how incorrect we can be in our parameter estimates while still meeting the constraints. Varying the parameters by increasing them will obviously continue to satisfy the constraints.

## Results

For the Keppel Islands case study, we identify an optimal reserve network that can simultaneously satisfy both the representation and demographic persistence constraints, and incurs a minimal opportunity cost on fishers while doing so. The optimal network designates 8 planning units covering 38% of the total reef area in the region as no-take reserves: 36%, 41%, and 36% of reef flat, crest and slope habitats respectively (Fig 1b). The network therefore exceeds the nominal constraints for feature representation (35% of each habitat), particularly for reef slope. In keeping with the spatially heterogeneous nature of larval dispersal, the optimal reserve network delivers widely different amounts of recruitment to the different planning units: one receives only 102% of its constraint, while another receives 110 times its constraint. Given that coral reef fish larval recruitment is believed to be space-limited [88], both of these planning units would contain comparable densities of adults, with the latter experiencing higher levels of compensatory mortality. The dual constraint reserve network satisfies these constraints by excluding fishers from planning units that contained 40% of the fishable biomass in the system.

The feature representation network (Fig 1c) is the same size as the dual constraint network, and protects almost exactly the constraint amount of the three reef habitat types (37%, 36% and 35% of reef flat, crest and slope habitat respectively), and therefore 36% of the total reef area in the Keppel Islands. Because it does not seek to achieve the recruitment constraints, two planning units in the system do not receive adequate levels of recruitment (they received 85% and 95% of the constraint). While this is not a large deficit, and while these planning units represent a small subset of the reserve network, this shortfall means that persistence cannot be guaranteed within all planning units. Reserved planning units experiencing a shortfall would no longer necessarily export as much larvae as expected, and this would compromise the persistence of all downstream reefs. The feature representation reserve network incurs a lower opportunity cost on fishers, excluding them from planning units that contain 36% of the fishable biomass in the system. The network designed to achieve only the recruitment constraints (Fig 1d) is different again. This network is much larger than the others—protecting 17 planning units—and provides very different habitat representation: 2%, 46% and 41% of reef flat, crest and slope habitats respectively, and 27% of the total reef habitat area. The network imposes an opportunity cost on recreational fishers that is approximately equivalent to the other networks.

We use the proportional overlap between the different reserve networks to assess whether satisfying the different constraints (representation-only, persistence-only, dual-constraint) requires different sets of planning units. The planning units chosen by the three optimal reserve networks are visibly different (Fig 1), but there is still some overlap. The persistence-only and representation-only reserve networks have an 11% overlap, as do the persistence-only and dual-constraint networks. However, this does not indicate that the planning units selected by multiple networks are particularly important, nor that the networks are significantly similar, since this amount of overlap is seen in random networks of the same size. Similarly, although the dual-constraint and representation-only networks have much less overlap (sharing only 3% of their planning units), given the smaller size of their networks, even this small amount of overlap is not significantly more or less than random expectation.

**Fig 2:**
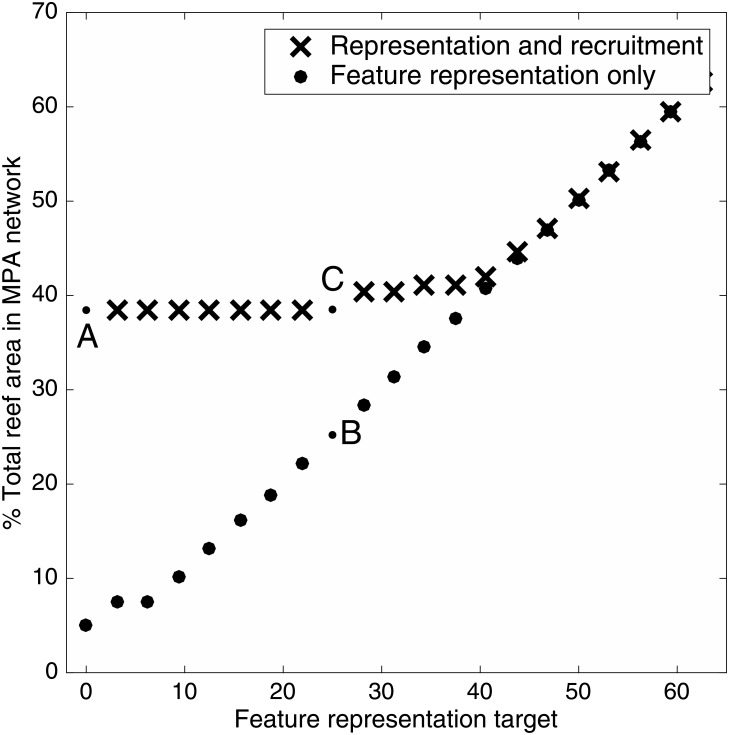
Relative strength of the two constraints on the conservation plan. The constraint for feature representation increases along the x-axis. Black crosses show the size of the no-take reserve networks required to optimally satisfy both constraints, with the demographic persistence constraints kept constant at our calculated values and the feature representation constraint increasing from left to right. Grey circles show the protection needed to optimally meet the constraints for feature representation, but not the constraint for demographic persistence. The marker ‘A’ is equivalent to the reserve network in Fig 1d. The marker ‘B’ is equivalent to Fig 1c. The marker ‘C’ is equivalent to Fig 1b.

The two sets of constraints can complement each other in the Keppel Islands (Fig 2). While feature representation constraints remain lower than 30%, the reserve network designed for persistence (marked ‘A’) does not need to change to accommodate increasing feature goals, since the requisite amount of feature representation is achieved in the course of ensuring the demographic constraints for recruitment. Only when the representation constraints exceed 40% of each feature does the reserve network begin to expand. Beyond this point, the dual constraint networks mirror the feature representation networks, indicating that recruitment constraints are likely being satisfied incidentally in the course of achieving the feature constraints. The figure can therefore be divided into two regimes: on the left, the dual constraint problem is reduced to a demographic persistence problem; on the right, the problem reduces to a feature representation problem. The marker indicated by ‘A’ on the extreme left of the figure is equivalent to the reserve network necessary to meet only the demographic persistence constraints (shown spatially in Fig 1d). The marker indicated by ‘B’ represents the reserve network designed only for habitat representation (shown spatially in Fig 1c). The marker indicated by ‘C’ represents the dual constraint network.

Optimal reserve networks—particularly those based on constraints—are notoriously sensitive to parameter uncertainty, since the design algorithms aim to meet the constraints as exactly as possible, in order to minimise the objectives, which are normally acting in opposition (e.g., minimise costs). We therefore explore the tolerance of our optimal network to pessimistic uncertainty in both the demographic parameters, and in the two different constraints. In general, the optimal reserve network is far more tolerant to pessimistic variation in the demographic persistence constraints and recruitment matrices, than it is to pessimistic variation in the feature representation and the distribution of habitat types. The optimal network is able to satisfy both sets of constraints while: (1) the increase in the feature targets remains within 1%; (2) the increase in the demographic persistence targets remains within 50%; (3) the decrease in the amount of habitat features in each planning unit remains within 1%; and (4) the decrease in the elements of the recruitment matrix remains within 21%. If the amount of variation is less than these amounts, the reserve network can still achieve both sets of conservation constraints for 95% of randomly altered systems.

From these results, it is clear that the optimal reserve design is far more sensitive to variation in the habitat features and targets. This outcome can seem counter-intuitive, since feature representation targets are far easier to achieve in the Keppels Islands case study than demographic persistence targets (Fig 2). However, feature representation has fewer dimensions than recruitment (i.e., there are only three habitat features, and hundreds of elements in the recruitment matrices), and features are more evenly distributed among the planning units than recruitment. As a result, the algorithm is generally able to find a reserve network that efficiently achieves the feature representation targets, with a negligible amount of surplus feature protection. While this makes the optimal reserve network efficient from a feature representation perspective, it means that changes to the network can easily compromise those targets. In contrast, demographic persistence targets are harder to meet perfectly, and therefore are often over-achieved. The networks are consequently more robust to uncertainty about dispersal.

The solution approach, implemented in Matlab’s binary programming function (*bintprog*; Matlab 2012), applies a linear-programming based branch-and-bound algorithm. On a desktop computer, this method identified the optimal solution for the Keppel Islands problem (36 planning units) in under a second. The running time of binary programming methods increases nonlinearly with problem complexity (the number of planning units in this case). Based on an analysis of systems with randomly generated features and dispersal patterns, applying our method to a problem with 50 planning units takes less than half a minute, while 100 planning units would take approximately 2 hours.

## Discussion

Here we advance a methodological approach that provides an explicit and mechanistic approach to adding demographic persistence constraints to standard feature representation systematic conservation planning approaches. The methods are specifically tailored for patchy marine ecosystems with demographically essential larval connectivity. Furthermore, our case study demonstrates that the required constraints for demographic persistence can be parameterized, albeit in a data-rich coral reef ecosystem. While systematic conservation planning strives to represent the full extent of biodiversity and also to ensure long-term persistence within managed areas [25], to date it has been more successful at the former goal than the latter. Our successful inclusion of population persistence into a systematic conservation planning framework addresses this issue and we illustrate how it can be applied in a data-rich context.

Prior to this work, the most common approaches to incorporating connectivity and demographic persistence into multi-species reserve network design—including marine reserve network design—were either conceptually unproven, or computationally challenging [43]. The first set of approaches incorporate qualitative design criteria associated with the spatial extent of connectivity into standard representation planning tools. These criteria primarily involve the size, shape, and spacing of no-take reserves. These methods are simple but, as we discuss in the introduction, are not explicitly linked to the persistence requirements of particular species [4,48]. It is therefore unclear if they would achieve their stated purpose without the application of separate, *post hoc* testing [89]. The second set of approaches are very explicit in their incorporation of ecological processes, including connectivity. Researchers are now creating decision-support tools that extend population viability analyses (PVAs) to incorporate spatial processes and conservation actions such as protected areas. These methods approach the problem of reserve design from the direction of population ecology, in contrast to the biogeographic origins of systematic conservation planning. The tools generally couple ecological niche modelling with stochastic population simulations [90–94], and are primarily applied to terrestrial conservation problems. Because they describe processes like connectivity explicitly and mechanistically, they are naturally suited to including connectivity information. However, while such approaches are mechanistic, consider uncertainty explicitly, and can be meaningfully parameterized [64,94], they are too computationally intensive to apply to the millions of possible reserve networks that need to be assessed in the process of systematic reserve network design. The methods are therefore best suited to assessing the relative performance of a small number of potential configurations in well-studied ecosystems (i.e., management strategy evaluation; Bunnefeld et al. 2011).

Our method occupies the space between these two approaches. It attempts to retain much of the practical simplicity that is the strength of the qualitative design criteria by expressing persistence (a dynamic process) as a set of static constraints (Eq 2). This approach allows persistence to be incorporated into the same well-tested optimization techniques as feature representation. However, like the spatial PVA approaches, our method is also based on a mechanistic description of population processes, and the constraints that it sets are explicitly linked to the overall conservation goals of demographic persistence. It is therefore able to make explicit and testable predictions about persistence, and to use ecological data to inform the concept of “adequate protection”. Because it offers an intermediate level of complexity and process, our method cannot displace either of the two existing alternatives. Qualitative design criteria (e.g., size and spacing rules) will still be required when decisions are needed with little information, a situation that applies across the tropical world, and for many critical coral reef fishery species even in developed economies. At the other extreme, spatially- and temporally-explicit PVAs will provide more accurate guidance when data and computational time are not limiting, and when only a small number of alternative scenarios need to be assessed. However, many situations exist between these two extremes, and methods such as ours therefore have the potential to provide useful and rapid decision-support in appropriate situations. Computationally, our method ran for the Keppels case study in under a minute. Although larger problems will take longer, heuristic methods such as simulated annealing (Kirkpatrick et al. 1983) or tabu search (Glover 1986) can help speed up the process.

The results from the Keppel Islands case study provide insights into two interesting elements of conservation planning for both feature representation and demographic persistence. First, less habitat was required to represent habitat in the Keppels than to ensure persistence (Fig 2). There are three reasons for this. First, in this case study the constraints for per-reef recruitment are more demanding than those for representation: 35% of the total amount of each feature must be protected, but as much as 85% of pristine recruitment (i.e., the recruitment that would occur without any fishing) is required on some reefs. Second, persistence constraints will almost always be much more numerous than representation constraints: each planning unit will have its own recruitment constraint, but features can generally be found in multiple locations. Finally, the persistence constraints are more interdependent than the representation constraints. Adding or removing a new marine reserve to satisfy the recruitment constraints of one planning unit will increase recruitment across the entire network. The dominance of recruitment constraints over representation constraints may not be true for every case study. Nevertheless, whenever conservation planning undertakes multi-constraint planning, one set of constraints is likely to be much harder to satisfy than the others. The conservation planning problem then simplifies to a problem addressing a single set of constraints—demographic persistence in the Keppel Islands case study—with the other set(s) being satisfied incidentally, in the process of satisfying the binding constraints (e.g. Fig 2). Once the binding constraints have been identified, the resulting simplified problem can potentially be solved with much less information, and with greater attention to the specifics of the more important process or pattern.

The second insight is that in dispersive environments such as marine ecosystems, the fundamental goals of conservation planning—that of representing a heterogeneous and multidimensional set of features, and then ensuring the persistence of those features—pull protected area networks in contrasting directions. Conservation features, such as habitats or species distributions, tend to be spatially autocorrelated, as a result of both autocorrelated environmental conditions, and the aggregative influence of local dispersal [10]. Scattered no-take reserves are the most efficient approach to producing a complementary reserve network in such environments, since reserves that are close together will provide redundant protection. In contrast, demographic persistence will often require reserves to be close together, so that they can offer mutual support through an exchange of recruits. Persistent reserve networks, particularly where anthropogenic pressures in surrounding non-reserve areas are strong, will therefore demand aggregated reserve configurations. Representation and persistence constraints will only be simultaneously satisfied if managers create large (i.e., expensive) reserve networks, which protect clusters of habitat throughout the entire system. Conservation plans that demand both representation and persistence are therefore not just expensive because they must satisfy more constraints, but also because the spatial configurations needed to achieve those constraints are in diametric opposition.

Although the Keppel Islands case study demonstrates that our method can be meaningfully parameterized for realistic conservation problems, some of the characteristics of the system limit our ability to draw broader conclusions from the results. First, the GBRMP in general, and *P*. *maculatus* in the Keppels specifically, represents a data-rich scenario that has been studied along numerous dimensions (e.g., demography, biogeography, dispersal ecology) and across lengthy timescales. Even so, the persistence constraints we constructed required multiple assumptions. To give three examples: First, we did not characterize the stochasticity that is known to drive dispersal at multiple timescales, nor did we consider changes in dispersal patterns that could result from coral reef habitat degradation [95] or climate change [3]. Second, the recruitment constraints were based on estimates of adult mortality that ignored density-dependent factors. Third, we were forced to estimate the amount of larval mortality indirectly, from the currently observed populations. Estimates of inter-patch dispersal were also essential, based on extensive genetic parentage analysis [24] that are currently available for only a handful of locations globally [96]. We stress that these data issues are not limitations of our method *per se*; they are questions that must be resolved if demographic persistence is to be explicitly included in conservation planning decisions. Indeed, they are central elements of the spatially-explicit metapopulation viability approach to including demographic persistence into conservation planning [64,94]. However, the data requirements will limit the locations where our method can be applied with confidence. In particular, our method will be most applicable to marine planning exercises across local or sub-regional extents, where data on demographic processes exist for a small number of key species, whose persistence is particularly threatened by direct anthropogenic activities such as fishing or habitat degradation.

By considering both the representation of biodiversity and the dispersal and persistence of a small set of key species, our method moves beyond many other contemporary analyses, as we describe above. However, at the same time we have used only simple conservation goals and conservation actions, compared to recent advances in marine reserve planning theory. First, the goals of conservation planning are increasingly broader than biodiversity representation and population persistence. This is particularly in coral reef ecosystems whose biodiversity and ecosystem processes support local economies and provide effectively irreplaceable food and coastal protection [97–100]. Limiting our demographic persistence constraint to a small number of species will inevitably bias the optimal reserve network towards that subset of biodiversity. This bias will only be acceptable to stakeholders and planners if the targeted species are disproportionately important: for example those highly valued by commercial or recreational fisheries, such as *P*. *maculatus*; or species of particular conservation concern such as the threatened humphead parrotfish *Bolbometopon muricatum* [101]; or species that can operate as umbrella species for a large range of others. Second, conservation actions are expanding beyond the long-term protection of habitat and populations, into marine reserves, into multiple use zones [102], habitat restoration [99], and dynamic protected status [46,103]. These actions better reflect the opportunities and limitations offered by local conditions, and can thereby achieve more efficient outcomes.

The inclusion of fishery species provides further rationale for taking a mechanistic approach to including demographic persistence, and also indicates how our extension of standard conservation planning techniques could be further expanded to better reconcile reserve network planning for biodiversity conservation and fisheries management [104]. The GBRMP zoning plan was conceived and implemented with the primary aim to conserve biodiversity, whilst also seeking to minimize negative impacts on reef users. Thus, our Keppel Islands case study minimized fisher’s opportunity costs while delivering sustainable recruitment. However, both of these components should be parameterized in ways that better reflect fishers’ expectations (e.g., their preference for large individuals), and which also incorporate some of the dynamical complexity of fisheries (e.g., displaced effort). Through more complex objectives, our approach could be used to maintain or increase fishery yields, while simultaneously ensuring the representation and persistence of key conservation features. Demographic persistence elements, like the ones included in our method, will need to be present in any conservation planning method that seeks to integrate these factors.

## Supporting Information

S1 Fig Recruitment matrix for the Keppel Islands case study. Visualisation of the 36 x 36 recruitment matrix for *Plectropomus maculatus* between the planning units of the Keppel Islands case study. Colors indicate the strength of dispersal between planning units. Note that the matrix is both heterogeneous and asymmetric.
(TIF)

S1 Table Habitat features in each planning unit. Numerical elements defining the feature matrix **M.** This table shows the transposed matrix, which has dimensions (3 x 36).
(DOCX)

S2 Table Recruitment matrix for the Keppel Islands case study, with unprotected source reefs. Numerical elements defining the recruitment matrix R*. The elements *r* (for the row) and *c* (for the column) of [R*]_**rc**_ show the number of larvae that would travel from an unprotected reef *r* to reef *c* (regardless of the protection status of reef *c*).
(XLSX)

S3 Table Recruitment matrix for the Keppel Islands case study, showing the additional recruitment that would come from protected source reefs. Numerical elements defining the recruitment matrix **R**. The elements *r* (for the row) and *c* (for the column) of [**R]**_**rc**_ shows the number of additional larvae that would travel from reef *r* to reef *c*, if reef *r* were protected, above the number that would travel between these two reefs in this direction if reef *r* were unprotected.
(XLSX)

S4 Table Constraint matrix for recruitment to each planning unit in the Keppel Islands case study. Numerical elements defining the recruitment constraint matrix **T**. Each value shows the number of larvae that need to settle on each reef in the system to ensure persistence if that planning unit is protected.
(DOCX)

S1 Text Constructing the binary programming problem. Supporting methods demonstrating that the optimisation algorithm can be constructed as a binary programming problem.
(DOCX)
