# Supplementary figures and images for: Planning Marine Reserve Networks for Both Feature Representation and Demographic Persistence Using Connectivity Patterns
Source: PLoS One. 2016 May 11;11(5):e0154272. doi: 10.1371/journal.pone.0154272 (PMC4864080; doi:10.1371/journal.pone.0154272)

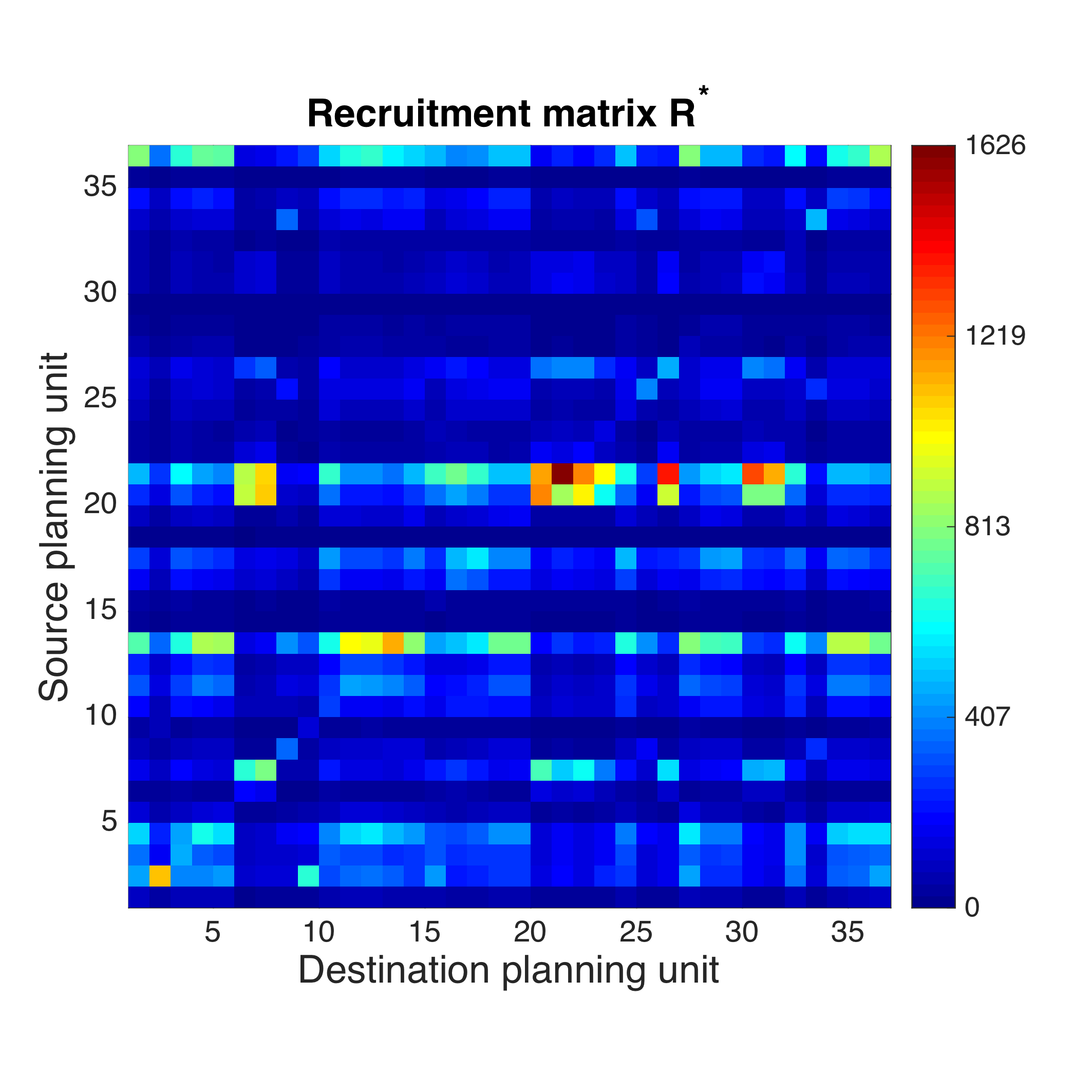

Supplement: S1 Fig — Visualisation of the 36 x 36 recruitment matrix for Plectropomus maculatus between the planning units of the Keppel Islands case study. Colors indicate the strength of dispersal between planning units. Note that the matrix is both heterogeneous and asymmetric. (TIF) [file pone.0154272.s001.tif]
