# Supplementary material for: Planning Marine Reserve Networks for Both Feature Representation and Demographic Persistence Using Connectivity Patterns
Source: PLoS One. 2016 May 11;11(5):e0154272. doi: 10.1371/journal.pone.0154272 (PMC4864080; doi:10.1371/journal.pone.0154272)
Supplement: S1 Table — Numerical elements defining the feature matrix M. This table shows the transposed matrix, which has dimensions (3 x 36). (DOCX) [file pone.0154272.s002.docx]

**SI Table 1: Habitat features in each planning unit**

Numerical elements defining the feature matrix **M.** This table shows the transposed matrix, which has dimensions (3 x 36).

| **Planning unit name** | **Crest** | **Flat** | **Slope** |
| --- | --- | --- | --- |
| *'Bald Rock'* | 0.91 | 2.43 | 0 |
| *'Barren Is'* | 12.98 | 26.2 | 1.89 |
| *'Big Peninsula'* | 6.87 | 10.21 | 1.61 |
| *'Clam Bay'* | 7.66 | 6.33 | 34.21 |
| *'Coconut Point'* | 2.5 | 3.27 | 0 |
| *'Conical Rocks'* | 2.79 | 4.31 | 0 |
| *'Corroboree Is'* | 7.57 | 18.23 | 6.78 |
| *'Divided Is'* | 5.47 | 7.73 | 0 |
| *'Egg Rock'* | 0.83 | 3.24 | 0 |
| *'Halftide Rocks'* | 2.11 | 7.77 | 0 |
| *'Halfway Is'* | 4.18 | 6.3 | 19.47 |
| *'Halfway Is (MPA)'* | 2.73 | 7.89 | 0.59 |
| *'Humpy Is'* | 8.56 | 20.56 | 36.97 |
| *'Long beach'* | 0.39 | 0.41 | 1.49 |
| *'Man and Wife'* | 0.92 | 1.57 | 0 |
| *'Miall Is'* | 4.22 | 7.62 | 5.28 |
| *'Middle Is'* | 8.41 | 7.4 | 20.55 |
| *'Monkey Point'* | 0.32 | 0.27 | 0 |
| *'Monkey Point (MPA)'* | 0.7 | 0.61 | 15.54 |
| *'North Keppel Is'* | 9.91 | 11.25 | 76.66 |
| *'North Keppel Is (East)'* | 21.51 | 38.16 | 0 |
| *'North Keppel Is (West)'* | 2.08 | 0.99 | 14.99 |
| *'Outer Rocks'* | 2.68 | 3.01 | 0 |
| *'Passage Rocks'* | 2.12 | 3.05 | 0 |
| *'Pelican Is'* | 6.44 | 8.26 | 2.12 |
| *'Pumpkin Is'* | 3.55 | 4.58 | 30.31 |
| *'Red Beach'* | 1.38 | 1.44 | 0 |
| *'Shelving Beach'* | 0.57 | 0.23 | 4.46 |
| *'Shelving Beach (MPA)'* | 0.44 | 0.21 | 0.17 |
| *'Sloping Is'* | 5.08 | 3.65 | 1.2 |
| *'Square Rocks'* | 2.41 | 5.15 | 0 |
| *'Svenson''s Beach'* | 3.08 | 0.67 | 0 |
| *'Wedge Is'* | 8.65 | 9.98 | 0.53 |
| *'Whitfield cove'* | 6.28 | 5.76 | 0 |
| *'Whitfield cove (MPA)'* | 0 | 0.79 | 0 |
| *'Wreck Bay'* | 7.92 | 22.26 | 0 |
