# Supplementary material for: Planning Marine Reserve Networks for Both Feature Representation and Demographic Persistence Using Connectivity Patterns
Source: PLoS One. 2016 May 11;11(5):e0154272. doi: 10.1371/journal.pone.0154272 (PMC4864080; doi:10.1371/journal.pone.0154272)
Supplement: S4 Table — Numerical elements defining the recruitment constraint matrix T. Each value shows the number of larvae that need to settle on each reef in the system to ensure persistence if that planning unit is protected. (DOCX) [file pone.0154272.s005.docx]

**SI Table 4: Constraint matrix for recruitment to each planning unit in the Keppel Islands case study.**

Numerical elements defining the recruitment constraint matrix **T**. Each value shows the number of larvae that need to settle on each reef in the system to ensure persistence if that planning unit is protected.

| **Planning unit name** | **Recruitment constraint (# larvae)** |
| --- | --- |
| *'Bald Rock'* | 460 |
| *'Barren Is'* | 5277 |
| *'Big Peninsula'* | 2234 |
| *'Clam Bay'* | 3030 |
| *'Coconut Point'* | 718 |
| *'Conical Rocks'* | 906 |
| *'Corroboree Is'* | 3786 |
| *'Divided Is'* | 1661 |
| *'Egg Rock'* | 583 |
| *'Halftide Rocks'* | 1407 |
| *'Halfway Is'* | 2143 |
| *'Halfway Is (MPA)'* | 1499 |
| *'Humpy Is'* | 5492 |
| *'Long beach'* | 158 |
| *'Man and Wife'* | 323 |
| *'Miall Is'* | 1767 |
| *'Middle Is'* | 2691 |
| *'Monkey Point'* | 69 |
| *'Monkey Point (MPA)'* | 799 |
| *'North Keppel Is'* | 5758 |
| *'North Keppel Is (East)'* | 7774 |
| *'North Keppel Is (West)'* | 944 |
| *'Outer Rocks'* | 690 |
| *'Passage Rocks'* | 653 |
| *'Pelican Is'* | 1910 |
| *'Pumpkin Is'* | 2269 |
| *'Red Beach'* | 338 |
| *'Shelving Beach'* | 267 |
| *'Shelving Beach (MPA)'* | 75 |
| *'Sloping Is'* | 1028 |
| *'Square Rocks'* | 1011 |
| *'Svenson''s Beach'* | 346 |
| *'Wedge Is'* | 2290 |
| *'Whitfield cove'* | 1409 |
| *'Whitfield cove (MPA)'* | 127 |
| *'Wreck Bay'* | 4176 |
