# Supplementary material for: Planning Marine Reserve Networks for Both Feature Representation and Demographic Persistence Using Connectivity Patterns
Source: PLoS One. 2016 May 11;11(5):e0154272. doi: 10.1371/journal.pone.0154272 (PMC4864080; doi:10.1371/journal.pone.0154272)
Supplement: S1 Text — Supporting methods demonstrating that the optimisation algorithm can be constructed as a binary programming problem. (DOCX) [file pone.0154272.s006.docx]

**SI Text: Constructing the binary programming problem**

It is relatively straightforward to convert the $P$ planning unit optimisation problem in the main text into the binary programming format:

$$\min_{N} \mathbf{G}N,$$

such that $\mathbf{A}N\leq c$.

Equation S1

The first line of this problem formulation states that the goal of the marine reserve network is to minimise the opportunity cost to fishers; the second line states that this goal has to be achieved while satisfying a set of constraints – specifically that both the representation of $F$ features and the persistence of $S$ species is achieved. $N$ is a $\left( P\times1 \right)$ binary control vector, where the values of $N_{i}$ indicate whether planning unit $i$ is protected ($N_{i}=1$) or unprotected ($N_{i}=0$),and $\mathbf{G}$ is a $\left( 1\times P \right)$vector containing the opportunity costs associated with each planning unit:

$$\mathbf{G}=\left[ \sum_{s} V_{s}b_{1,s}, \sum_{s} V_{s}b_{2,s}, \ldots, \sum_{s} V_{s}b_{i,s},\ldots,\sum_{s} V_{s}b_{P,s} \right].$$

Equation S2

The parameter $V_{s}$ is the relative value to fishers of each species, and $b_{i,s}$ is the abundance of each species $s$ found in each planning unit $i$ when that planning unit is unprotected (i.e., fished), calculated using the surveyed fish density in the different habitat types, and the amount of those habitat types in each planning unit (stored in the feature matrix in our problem).

$\mathbf{A}$ is an $\left( \left( F+SP \right)\times P \right)$ block constraint matrix composed of the feature matrix $\mathbf{M}$ and the protected recruitment matrices corresponding to the $S$ species being protected (see main text for an explanation of both, and *Supplementary Table 1* for the feature matrix for the Keppels Island group). These recruitment matrices are transposed as they sum the recruitment into, rather than out of, each planning unit:

$$\mathbf{A}=\left[ \begin{matrix} \left[ \mathbf{M} \right] \\ \left[ \mathbf{R}_{\mathbf{1}} \right]^{T} \\ \begin{matrix} \left[ \mathbf{R}_{\mathbf{2}} \right]^{T} \\ \vdots\\ \left[ \mathbf{R}_{\mathbf{S}} \right]^{T} \end{matrix} \end{matrix} \right].$$

Equation S3

Finally, $c$ is an $\left( \left( F+P \right)\times1 \right)$ constraint vector:

$$c=\left[ \begin{matrix} \left[ \mathbf{Q} \right] \\ \left[ \mathbf{U}_{1} \right] \\ \begin{matrix} \left[ \mathbf{U}_{2} \right] \\ \vdots\\ \left[ \mathbf{U}_{S} \right] \end{matrix} \end{matrix} \right],$$

Equation S4

In **Equation S4**, the matrix $\mathbf{Q}$ is a $\left( F\times1 \right)$ vector of total area targets for each feature in the system (i.e., across the whole reserve network). The $\mathbf{U}_{s}$ matrices are defined using the $T_{i,s}$ variables from §3.1 in the main text:

$$\mathbf{U}_{s}=\left[ \begin{matrix} T_{1,s}-\sum_{j} \left[ R_{s}^{*} \right]_{j1} \\ T_{2,s}-\sum_{j} \left[ R_{s}^{*} \right]_{j1} \\ \begin{matrix} \vdots\\ T_{P,s}-\sum_{j} \left[ R_{s}^{*} \right]_{j1} \end{matrix} \end{matrix} \right].$$

Equation S5

The elements of the block matrices $\mathbf{U}_{\mathbf{s}}$ can be understood as the amount of recruitment that the MPA network must deliver to each patch, for each species, above the level that would be provided by a completely unprotected metapopulation.

This problem can be readily solved using standard optimisation methods. We use Matlab’s (2012) binary programming implementation, which applies a linear programming branch-and-bound algorithm (Hillier et al. 2004). These techniques are very commonly used in operations research, and have previously been applied to large conservation planning problems (Crossman and Bryan 2006). For larger problems, heuristic methods can be used that search the solution space randomly but intelligently (e.g., genetic algorithms or simulated annealing), preferably from a large number of initial guesses to better identify the global optimum.

**References cited:**

Crossman, N. D., and B. A. Bryan. 2006. Systematic landscape restoration using integer programming. Biological Conservation 128:369–383.

Hillier, F. S., G. J. Lieberman, F. Hillier, and G. Lieberman. 2004. MP Introduction to Operations Research.
